# Sedentary Behaviour Impairs Skeletal Muscle Repair Modulating the Inflammatory Response

**DOI:** 10.3390/jfmk7040076

**Published:** 2022-09-27

**Authors:** Eduardo Teixeira, Juliana Garcia, António Bovolini, Ana Carvalho, Júlio Pacheco, José A. Duarte

**Affiliations:** 1 Research Center in Physical Activity, Health and Leisure (CIAFEL), Faculty of Sports, University of Porto (FADEUP) and Laboratory for Integrative and Translational Research in Population Health (ITR), 4200-450 Porto, Portugal; 2 Faculty of Psychology, Education and Sports, Lusófona University of Porto, 4000-098 Porto, Portugal; 3 Escola Superior Desporto e Lazer, Instituto Politécnico de Viana do Castelo, 4960-320 Melgaço, Portugal; 4 Centre for the Research and Technology of Agro-Environment and Biological Sciences (CITAB), Institute for Innovation, Capacity Building and Sustainability of Agri-Food Production (Inov4Agro), University of Trás-os-Montes e Alto Douro, 5000-801 Vila Real, Portugal; 5 AquaValor-Centro de Valorização e Transferência de Tecnologia da Água-Associação, 5400-342 Chaves, Portugal; 6 Instituto Politécnico da Guarda, Unidade de Investigação para o Desenvolvimento do Interior (UDI), 6300-559 Guarda, Portugal; 7 Research Center in Sports Sciences, Health Sciences and Human Development (CIDESD), University Institute of Maia, 4475-690 Maia, Portugal; 8 Toxicology Research Unit (TOXRUN), University Institute of Health Sciences (IUCS), Advanced Polytechnic and University Cooperative (CESPU), CRL, 4585-116 Gandra, Portugal

**Keywords:** rats, cardiotoxin, neutrophils, macrophages, regeneration, fibrosis, physical conditioning, motor activity

## Abstract

This study investigated whether sedentary behaviour modulates skeletal-muscle repair and tissue inflammatory response after cardiotoxin (CTX)-induced injury. Singly caged rats spent 8 weeks either as a sedentary group (SED, *n* = 15) or as a control group (EX, *n* = 15)—caged with running wheels for voluntary running. All rats had each tibial anterior muscle infused either with CTX (CTX; right muscle) or saline solution (Sham; left muscle) and were sacrificed (*n* = 5 per group) on the 1st, 7th, and 15th day post-injection (dpi). Histological and immunohistochemical analyses were used to calculate myotube percentage and fibrosis accretion, and quantify the number of neutrophils and M1 and M2 macrophage subtypes. The SED group showed an increased number of both neutrophils and M1 macrophages (7th and 15th dpi) compared to the EX group (*p* < 0.01). The EX group showed an increased number of M2 macrophages on the 1st dpi. On the 7th dpi, the SED group showed a lower myotube percentage compared to the EX group (*p* < 0.01) and on the 15th dpi showed only 54% of normal undamaged fibres compared to 90% from the EX group (*p* < 0.01). The SED group showed increased fibrosis on both the 7th and 15th dpi. Our results show that sedentary behaviour affects the inflammatory response, enhancing and prolonging the Th1 phase, and delays and impairs the SMR process.

## 1. Introduction

Sedentary behaviour, seen as regular prolonged periods of skeletal-muscle inactivity, not only induces multiple-organ dysfunction [1], but is also associated with all-cause mortality in adults [2]. Compelling evidence shows that permanent physical inactivity induces negative effects on the skeletal-muscle phenotype, affecting its morphology, composition, and function [3], but less is known regarding its plausible interference on the skeletal-muscle regenerative capacity and the tissue inflammatory response upon injury. The inflammatory response to skeletal-muscle injuries comprises intricate cellular and molecular interactions between both the skeletal muscle and the inflammatory cells [4]. Generally, upon muscle injury, the inflammatory response is characterized by a pro-inflammatory (or Th1) phase, mostly performed by neutrophils and pro-inflammatory macrophages (M1) that, despite causing further tissue damage, effectively stimulate the satellite-cell activation and proliferation stages. This Th1 phase is followed by an anti-inflammatory (or Th2) phase, performed by anti-inflammatory macrophages (M2), which reduces the Th1 phase by promoting inflammatory resolution and stimulates satellite-cell terminal differentiation and myoblast growth and maturation, supporting tissue repair [4,5]. This important complex cellular relationship influences skeletal-muscle repair (SMR) by modulating the function of the myogenic stem-cell population, which is fundamental to coping appropriately with different insults and to promoting successful muscle repair [6]. In fact, both the importance of the neutrophil contribution to a successful muscle-regenerative response [7] and the negative impact of an impaired macrophage polarization during SMR [8,9] have already been demonstrated. Among several factors influencing SMR (e.g., age, genetics, insult severity and nature) it is known that different patterns of skeletal-muscle recruitment may modulate its regenerative capacity. Indeed, increased skeletal-muscle loading, through increased physical activity or exercise training, seems to be beneficial in improving myogenic-cell functionality, muscle regeneration, and impairing fibrosis during SMR [10]. Moreover, acute muscle recruitment seems to alter macrophage functions, e.g., increasing their phagocytic activity [11], as well as their tumour cytotoxicity and chemotaxis capacities in both acute and chronic loading conditions [12]. However, studies addressing the effects of different levels of muscle recruitment, achieved through imposed sedentary behaviour, or increased voluntary physical activity, on the SMR process remain unexplored.

Considering that skeletal muscles are frequently exposed to various stressful conditions (e.g., day-to-day mechanical, thermic, and metabolic stress) and that a decreased capacity to perform muscular work increases predisposition to diseases and impairs quality of life [3], it seems relevant to acknowledge whether sedentary behaviour influences SMR, i.e., the process that maintains the skeletal-muscle quality and function. Therefore, the aim of this study was to test the hypothesis that sedentary behaviour impairs SMR, determining whether 8 weeks of decreased muscle recruitment alters the local inflammatory response and the healing process on the rat tibial anterior muscle in response to cardiotoxin (CTX)-induced damage.

## 2. Materials and Methods

### 2.1. Animals and Experimental Design

After arrival, 30 2-month-old male Wistar rats (Charles River Laboratories, Barcelona, Spain) were individually caged and kept in a controlled environment (constant humidity of 50–60% and temperature of 22 ± 2 °C) and in an inverted 12 h light/dark cycle. Standard rat chow and tape water were provided ad libitum during the entire protocol. Following one week of quarantine, rats were randomly assigned to either a sedentary group (SED, *n* = 15) or an exercise control group (EX, *n* = 15). EX rats, housed individually in cages equipped with a complete activity-wheel system (Tecniplast 2154F0105; overall dimensions of 480 × 315 × 470 mm^3^), were able to run voluntarily. SED rats, also housed individually in similar cages but without the activity-wheel system, were forced into sedentary behaviour, as previously carried out in our laboratory [13]. After 8 weeks in these conditions, each animal, under light anaesthesia induced by an intraperitoneal (ip) injection of ketamine and xylazine, had his right tibial anterior muscle injected with 30 μL of 20 μM cardiotoxin (CTX; Naja mossambica mossambica; C9759, Sigma-Aldrich, St. Louis, MO, USA) in the mid-belly portion (CTX muscle) and his left tibial anterior muscle injected with 30 μL of saline solution (Sham muscle) in the same muscle region. Lyophilized CTX venom was prepared by liquefying a recently opened container in PBS. The fresh solution was divided into Eppendorf tubes as 10 mL aliquot stocks, flash frozen, and stored at −80 °C. Each tube was thawed fresh before injection and not re-used. Injections were executed using a 50 μL, 22-gauge needle-fixed syringe (Hamilton; 20736, Sigma-Aldrich, St. Louis, MO, USA), and to preserve the experimental procedure consistency, all the injections were completed by the same researcher, certifying that similar infiltrations were carried out for all groups. Subsequently, all animals were kept in the same conditions and 5 animals from the SED and EX groups were sacrificed on the 1st, 7th, and 15th day post-injury (dpi). Total distance run by each rat of the EX group was documented daily. The study was approved by the ethics committee of the Faculty of Sport from the University of Porto (FADEUP - protocol code number 22-3/456/2014, approved on 16 April 2014). All the institutional guidelines [14] for the care and use of animals were followed.

### 2.2. Tissue Collection and Processing for Light Microscopy

All animals were sacrificed by a deadly ip injection of ketamine and xylazine and the tibial anterior muscles removed, cleansed of contiguous soft and fat tissue, cut in half in the mid-belly region, and fixated in a solution containing 4% paraformaldehyde (P6148; Sigma-Aldrich, St. Louis, MO, USA), 2.5% sucrose (S0389; Sigma-Aldrich, St. Louis, MO, USA), and 0.1% glutaraldehyde (G5882; Sigma-Aldrich, St. Louis, MO, USA) in PBS (pH 7.2) at 4 °C for 24 h. After fixation, both halves were dehydrated through graded-ethanol solutions, cleared in xylene, and embedded in paraffin blocks. Transverse 5 μm thick sections from each muscle part were cut on a Leica 2125 rotary microtome (Leica Microsystems, Wetzlar, Germany). The created slides were then used for muscle histomorphometry, evaluation of fibrotic-tissue accretion, and immunohistochemistry analysis.

### 2.3. Histomorphometry, Fibrosis, and Immunohistochemistry Analysis

For histomorphometry, muscle sections were stained with haematoxylin and eosin (H&E), examined under a light microscope (Axio Imager A1, Carl Zeiss; Jena, Germany) with a 40× magnification objective, and images were snapped by an attached digital camera with Axio Vision 4.7 software. The obtained non-overlapping images were then analysed with ImageJ software (NIH, Bethesda, Rockville, MD, USA) to quantify the morphological features of the muscle sections. For consistency, the histomorphological evaluation was accomplished on multiple single-frame images (5 to 10 images obtained per section) acquired from the same muscle section (9 different sections analysed per muscle) and, exclusively, within the damaged area, i.e., the muscle area with clear inflammatory infiltrate, the presence of necrotic fibres (degenerating fibres with fragmented sarcoplasm), regenerating fibres (small basophilic and central nucleated fibres with normal sarcoplasm), or excessive fibrosis. The level of CTX-induced muscle damage was assessed by calculating the percentage of necrotic fibres from the total number of analysed cells in each image during the 1st dpi. The SMR progress was evaluated by counting the percentage of myotubes (small basophilic and central nucleated fibres with normal sarcoplasm) and the fibrotic-tissue accretion at each dpi. The percentage of myotubes and normal fibres, i.e., fibres with peripheral nuclei, intact sarcolemma, and non-fragmented sarcoplasm, was determined from the total number of analysed cells in each image.

For fibrosis evaluation we applied the picrosirius red (SR)-staining method [15]. Briefly, slides were incubated on 0.1% SR in saturated picric acid for 1 h, rinsed in 0.5% acetic acid, dehydrated in ethanol, and cleared in xylene. This technique stains collagen tissue bright red and muscle tissue yellow. Again, 5 to 10 non-overlapping single-frame images, acquired from the same muscle section and within the damaged area, were examined with Image-Pro Plus 6.0 software (Media Cybernetics, Singapore). The area covered by fibrosis was quantified in all experimental groups.

The immunohistochemistry analysis was performed, as described by others [16], to quantify M1 and M2 macrophage subtypes and neutrophils present in the damaged-muscle area. Briefly, slides were deparaffinized and put in a pressure cooker for 20 min in 10 mM citrate buffer, pH 6.0, for the antigen-retrieval procedure. After cooling and washing twice with PBS solution for 5 min, the endogenous-peroxidase activity was blocked for 30 min with a fresh 3% solution of hydrogen peroxide in methanol. The non-specific binding sites were blocked with 3% bovine serum albumin (BSA) for 30 min. Following the blocking step, each slide was incubated with antibodies targeted to M1 and M2 macrophage subtypes and neutrophils, anti-CD68 antibody (ab125212; abcam, Cambridge, UK), anti-mannose receptor antibody (ab64693; abcam, Cambridge, UK), and anti-neutrophil elastase antibody (ab 21595; abcam, Cambridge, UK), all diluted (1:100) in PBS-T overnight (4 °C). Sections were washed with PBS and then probed with goat anti-rabbit IgG horseradish peroxidase secondary antibody (1:200) (ab97051; abcam, Cambridge, UK) in PBS-T for 2 h at 37 °C. The sections were then washed twice under gentle stirring with PBS for 5 min and incubated with 3,3′-diaminobenzidine tetrahydrochloride (DAB) reagent (D0426; Sigma-Aldrich, St. Louis, MO, USA) for 3 min. After washing, the slides were then counterstained with a solution of hematoxylin diluted in water (1:15) for 3 min. As a negative control, additional sections were treated similarly; however, the primary or secondary antibodies were substituted by PBS. Finally, slides were mounted, cover slipped, and analysed by light microscopy as already described. Additionally, since anti-CD68 antibody also reacted with early-developing myotubes, their total number within the analysed area was also measured. This procedure, together with the classic evaluation of central nucleated fibres with H&E staining, allowed different phases of myotube evolution to be distinguished.

### 2.4. Statistical Analysis

To determine within-group normality for a given variable, the Kolmogorov–Smirnov test was performed and the Levene’s test was performed to determine homogeneity of variance. Normal distributed variables are presented as mean and standard deviation. However, considering that most variables lacked normal distribution, the differences between groups and the differences on each dpi within groups were tested with the Mann–Whitney (two-tailed) test and the Kruskal–Wallis test, respectively. These variables are presented as median and interquartile range (IQR: 1st–3rd quartiles). SPSS version 21 (IBM, Armonk, NY, USA) was used to perform all statistical analyses. Statistical significance was accepted at *p* < 0.05.

## 3. Results

### 3.1. Body Weight and Voluntary Physical Activity

SED body weight was significantly higher than EX (417.6 ± 35.5 g vs. 384.4 ± 26.8 g, respectively, *p* < 0.01). During the 8 weeks, the EX group ran on average 19 ± 12.4 km/week.

### 3.2. Histomorphometry, Fibrosis, and Immunohistochemistry Analysis

**Figure 1:**
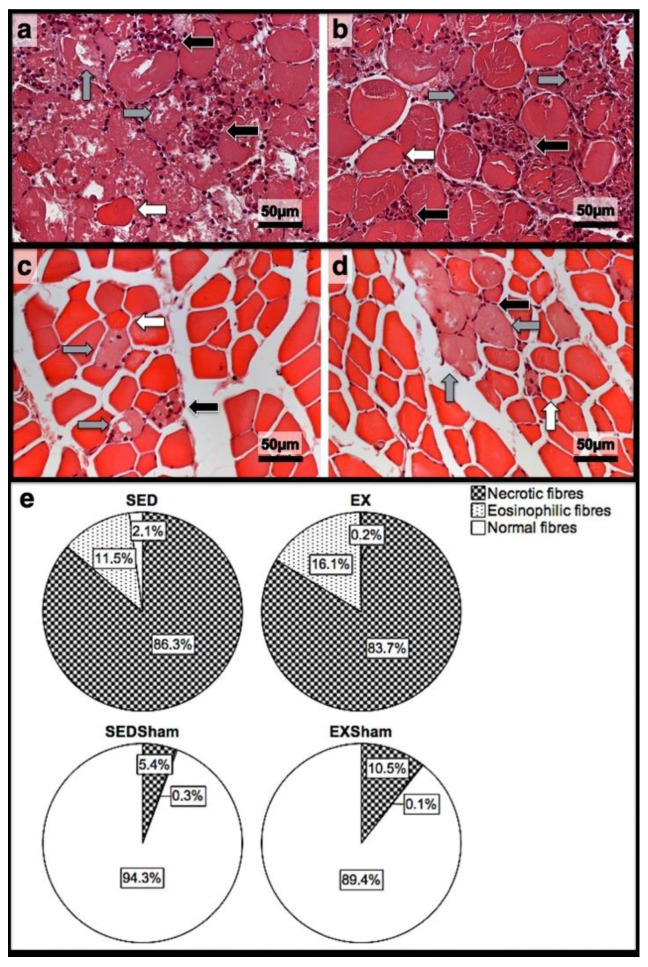
Representative images of the infused area of tibial anterior muscles stained with H&E from exercise (EX) and sedentary (SED) groups injected with cardiotoxin (**a**,**b**) and saline solution (Sham; (**c**,**d**)) and sacrificed on the 1st day post-injection. In (**e**), the corresponding quantitative analyses of necrotic (fibres with fragmented and infiltrated sarcoplasm), eosinophilic (hypercontracted, enlarged, and rounded fibres with eosinophilic staining, i.e., bright-red sarcoplasm), and normal (fibres with peripheral nuclei, intact sarcolemma, and non-fragmented sarcoplasm) fibres is depicted. In (**a**,**b**), a vast inflammatory infiltrate (black arrows), the existence of several necrotic fibres (grey arrows), and the existence of eosinophilic fibres (white arrows) is clear. On (**c**,**d**), despite the presence of some necrotic fibres (grey arrows), inflammatory infiltrate (black arrows), and fewer eosinophilic fibres (white arrows), the majority are normal fibres. EX—exercise group injected with cardiotoxin; SED—sedentary group injected with cardiotoxin; EXSham—exercise group injected with saline solution; SEDSham—sedentary group injected with saline solution.

**Figure 2:**
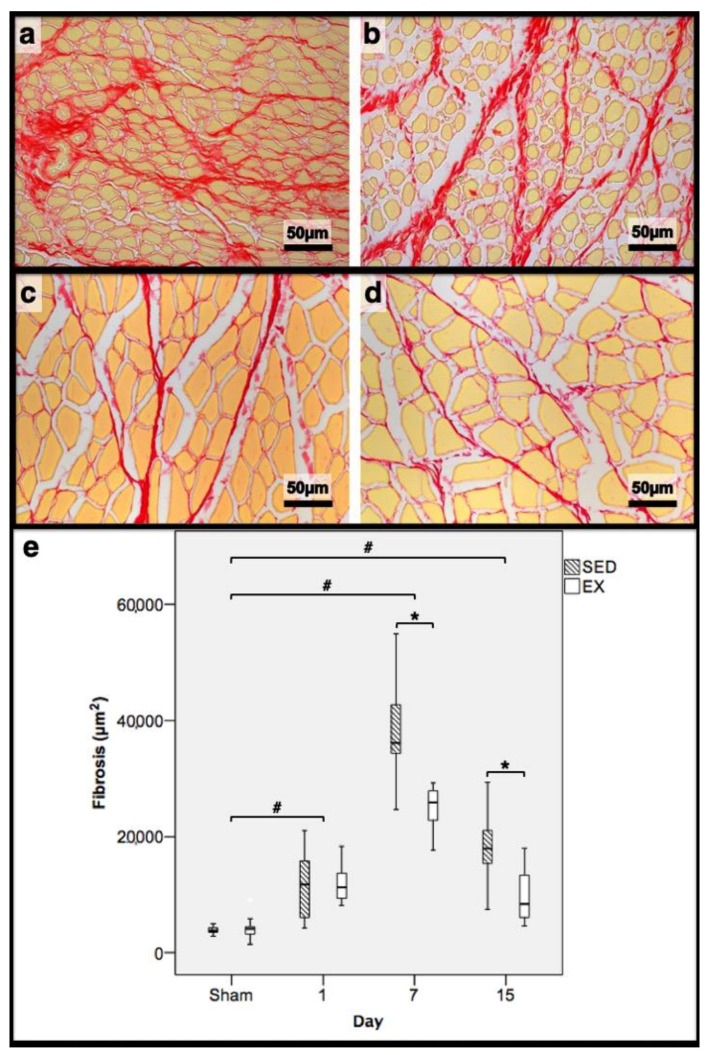
Representative images of the tibial anterior muscles, stained with picrosirius red, from the exercise (EX; (**a**,**c**)) and sedentary (SED; (**b**,**d**)) groups injected with cardiotoxin, sacrificed on the 7th (**a**,**b**) and 15th (**c**,**d**) day post-injection. In (**e**), the corresponding distribution of the fibrotic tissue area in all assessed days in both groups is depicted (Sham: overall median values of control muscles). Box = median, 25 to 75%; T-bars = minimum and maximum values. * significantly higher than EX group (*p* < 0.01); # significantly higher than Sham muscles (*p* < 0.01). SED—sedentary group injected with cardiotoxin; EX—exercise group injected with cardiotoxin; Sham—control muscles injected with saline solution.

**Figure 3:**
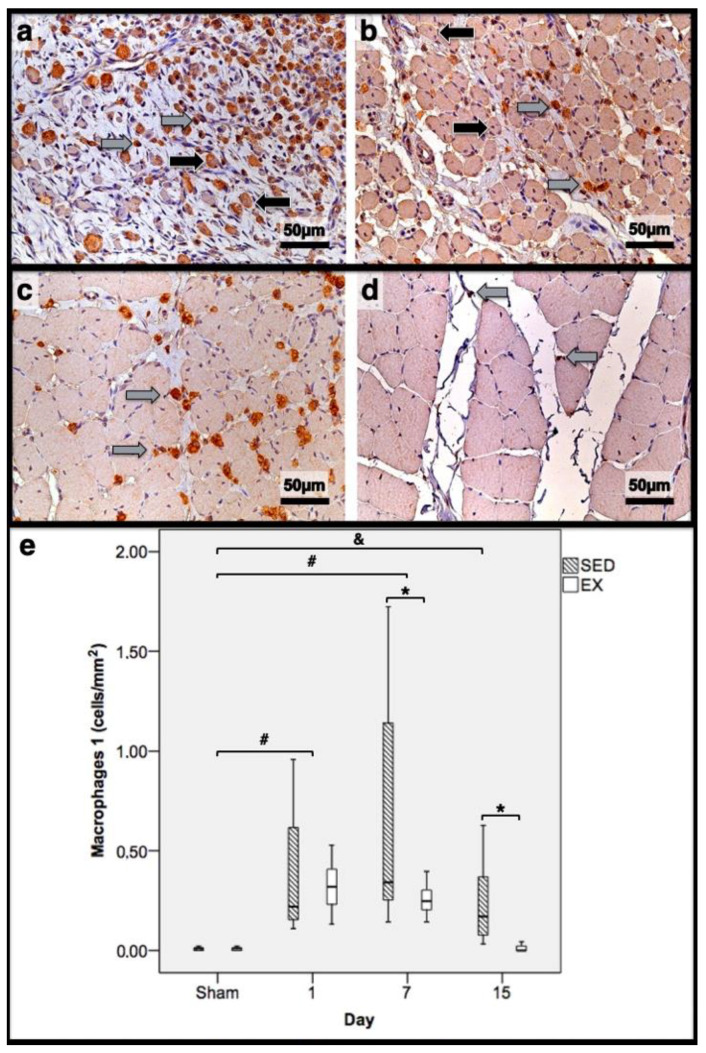
Immunohistochemistry of macrophages 1 (expressing CD68) in the tibial anterior muscles from the exercise (EX: (**a**,**c**)) and sedentary (SED: (**b**,**d**)) groups injected with cardiotoxin, sacrificed on the 7th and 15th day post-injection ((**a**,**b**) and (**c**,**d**), respectively), and corresponding quantitative analyses (**e**) in all assessed days in both groups (Sham: overall median values of control muscles). Sham muscles in both EX and SED, injected with saline solution, represent the values obtained on the 1st day post-injection. The images clearly show the brown-stained M1 macrophage (grey arrows) infiltration, which is more pronounced in the SED group (**b**,**d**). Additionally, many small myotubes with central nuclei are also observed, mainly in (**b**), expressing CD68 (black arrows). Box = median, 25 to 75%; T-bars = minimum and maximum values. * significantly higher than the EX group (*p* < 0.01); # significantly higher than the Sham muscles (*p* < 0.01); & SED group significantly higher than the Sham muscles (*p* < 0.01). SED—sedentary group injected with cardiotoxin; EX—exercise group injected with cardiotoxin; Sham—control muscles injected with saline solution.

**Figure 4:**
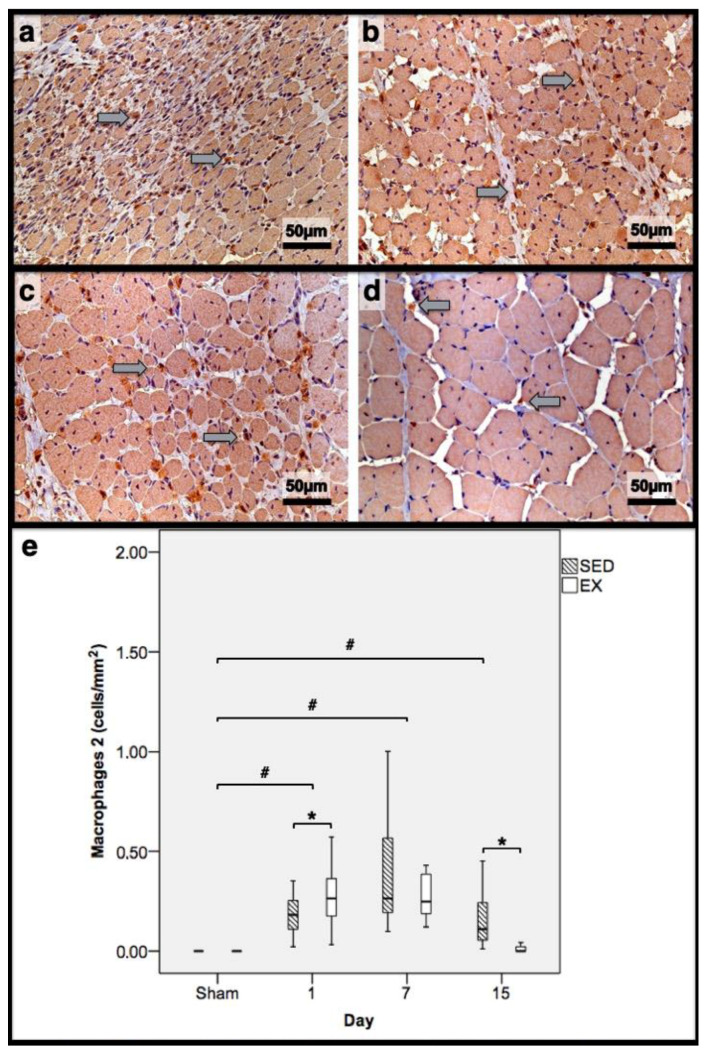
Immunohistochemistry of macrophages 2 (expressing mannose receptor) in the tibial anterior muscles from the exercise (EX: (**a**,**c**)) and sedentary (SED: (**b**,**d**)) groups injected with cardiotoxin, sacrificed on the 7th and 15th day post-injection ((**a**,**b**) and (**c**,**d**), respectively), and corresponding quantitative analyses (**e**) in all assessed days in both groups (Sham: overall median values of control muscles). Sham muscles in both EX and SED, injected with saline solution, represent the overall values obtained on the 7th day post-injection. The images clearly show the brown-stained M2 macrophage (grey arrows) infiltration, which is more pronounced in the SED group (**b**,**d**). Box = median, 25 to 75%; T-bars = minimum and maximum values. * significantly higher than the EX group (*p* < 0.01); # significantly higher than the Sham muscles (*p* < 0.01). SED—sedentary group injected with cardiotoxin; EX—exercise group injected with cardiotoxin; Sham—control muscles injected with saline solution.

**Figure 5:**
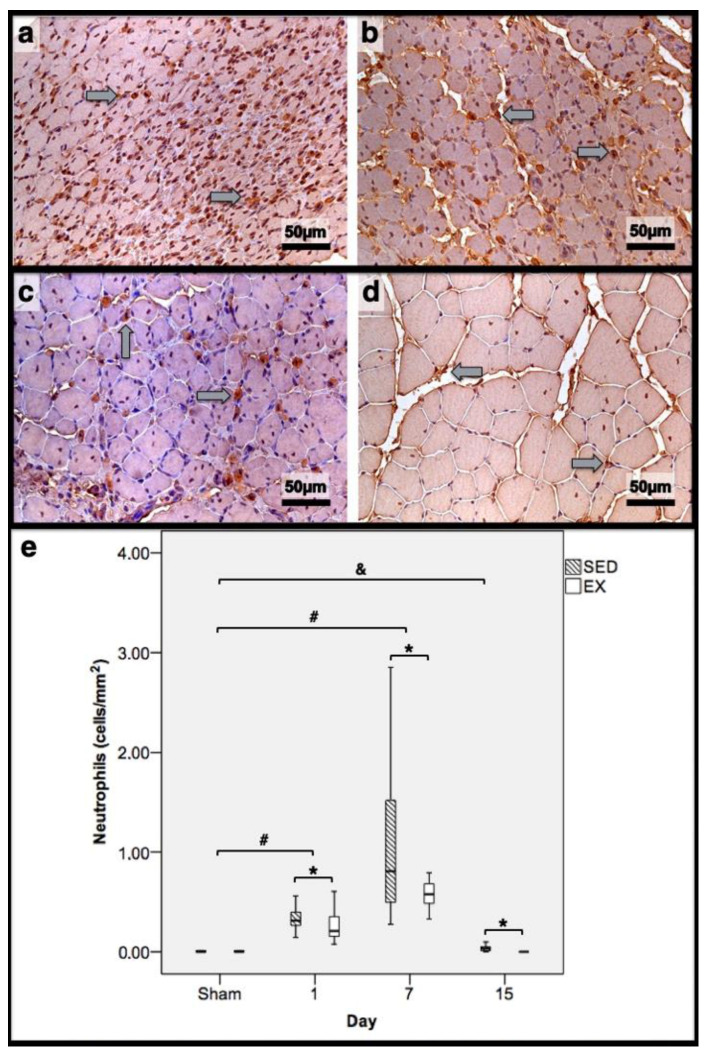
Immunohistochemistry of cells expressing neutrophil elastase in the tibial anterior muscles from exercise (EX: (**a**,**c**)) and sedentary (SED: (**b**,**d**)) groups injected with cardiotoxin, sacrificed on the 7th and 15th day post-injection ((**a**,**b**)and (**c**,**d**), respectively), and corresponding quantitative analyses (**e**) in all assessed days in both groups (Sham: overall median values of control muscles). Sham muscles in both EX and SED, injected with saline solution, represent the overall values obtained on the 1st day post-injection. The images clearly show the brown-stained neutrophil (grey arrows) infiltration, which is more pronounced in the SED group (**b**,**d**). Box = median, 25 to 75%; T-bars = minimum and maximum values. * significantly higher than the EX group (*p* < 0.01); # significantly higher than the Sham muscles (*p* < 0.01); & SED group significantly higher than the Sham muscles (*p* < 0.01). SED—sedentary group injected with cardiotoxin; EX—exercise group injected with cardiotoxin; Sham—control muscles injected with saline solution.

As illustrated in Figure 1, injections with CTX produced evident skeletal-muscle damage in both groups when compared to Sham muscles, which showed very restricted areas of muscle damage, possibly produced by the needle mechanical injury. To accurately examine the progression of the inflammatory cells’ infiltration and the development of fibrotic-tissue accretion throughout the experimental period in CTX muscles, comparisons were made with data obtained from Sham muscles. Images illustrating tibial anterior muscle sections stained with SR observed in all groups and related fibrosis-area distribution are depicted in Figure 2. Representative images of immunohistochemistry from M1 and M2 macrophages, neutrophils in the tibial anterior muscle sections, and corresponding quantitative analyses are illustrated in Figure 3, Figure 4 and Figure 5, respectively.

### 3.3. First Day Post-Injury

In the infused region, the EX and SED groups showed similar percentages of necrotic fibres (84% vs. 86%, respectively; *p* > 0.05); no presence of myotubes, as expected (Figure 1); and, as depicted in Figure 2, similar quantities of fibrotic-tissue area (11.7 (5.9–15.9) mm^2^ and 11.1 (9.3–13.8) mm^2^ for the SED and EX groups, respectively, *p* > 0.05). Both the EX and SED groups showed a similar number of M1 macrophages, as illustrated in Figure 3 (*p* > 0.05). The SED group showed a decreased number of M2 macrophages (Figure 4) compared to the EX group (*p* < 0.01). Finally, the SED group showed an increased number of neutrophils (Figure 5) compared to the EX group (*p* < 0.01).

### 3.4. Seventh Day Post-Injury

The SED group presented a decreased percentage of myotubes compared to the EX group (58.4% vs. 69.3%, respectively, *p* < 0.01), and, as depicted in Figure 2, increased fibrosis (36.1 (34.1–43.4) mm^2^ vs. 24.9 (22.3–28.3) mm^2^ for the SED and EX groups, respectively, *p* < 0.01). The SED group showed an increased number of M1 macrophages (Figure 3) compared to the EX group (*p* < 0.01). Additionally, the SED group showed an increased number of developing myotubes—detected by immunohistochemistry, as illustrated in Figure 3—compared to the EX group (5.5 (2–15.8) vs. 0 (0–1.8), respectively, *p* < 0.01). Both the EX and SED groups showed similar number of M2 macrophages, as depicted in Figure 4 (*p* > 0.05). Finally, the SED group showed an increased number of neutrophils (Figure 5) compared to the EX group (*p* < 0.01).

### 3.5. 15th Day Post-Injury

The SED group presented an increased percentage of myotubes compared to the EX group (46% vs. 10.2%, respectively, *p* < 0.01). Again, as illustrated in Figure 2, the SED group showed increased fibrosis compared to the EX group (17.9 (15.4–21.3) mm^2^ vs. 8.4 (6–14) mm^2^, respectively, *p* < 0.01). The SED group showed an increased number of M1 and M2 macrophages and neutrophils (as illustrated in Figure 3, Figure 4, and Figure 5, respectively) compared to the EX group (*p* < 0.01).

Briefly, as depicted in Figure 2, both the SED and EX groups showed an increased area of fibrosis throughout the experimental period and an increased number of M2 macrophages (Figure 4) compared to Sham muscles (*p* < 0.01). Regarding the number of M1 macrophages (Figure 3) and neutrophils (Figure 5) on the 1st and 7th dpi, both the SED and EX groups presented increased numbers of these inflammatory cells (*p* < 0.01); however, only the SED group showed augmented numbers on the 15th dpi, compared to Sham muscles (*p* < 0.01). Table 1 summarizes the overall histomorphometry and immunohistochemistry findings.

## 4. Discussion

Our results demonstrate that sedentary behaviour (1) deeply affects the local inflammatory cellular response upon injury, enhancing and prolonging the Th1 phase, and (2) delays/impairs the SMR process, as indicated by the number of myotubes and the fibrosis accretion throughout the experimental period.

On the 1st dpi, CTX-induced damage was similar between SED and EX groups, which presented no differences in the percentage of necrotic fibres. These results demonstrate that the injection protocol was homogenous between groups and that different patterns of muscle recruitment before the damage protocol did not change the vulnerability to CTX-related effects. Despite the similar number of M1 macrophages, the SED group showed an increased number of neutrophils compared to the EX group, suggesting that sedentary behaviour may make inflammatory cells prone to rapidly endorsing the Th1 response. Data from M2 macrophages seem to corroborate this hypothesis. Interestingly, considering the importance of the tissue microenvironment and the extrinsic factors, e.g., the surrounding cytokines, in governing macrophage polarization [17], it is appealing to consider that increased muscle recruitment before and during the SMR acutely stimulates macrophages to polarize into an anti-inflammatory state. In fact, the EX group showed an increased number of M2 macrophages compared to the SED group only 24 h post-injury.

On the 7th dpi, the SED group developed an apparent unfavourable response, showing a lower myotube percentage, an increased number of developing myotubes (indicative of a delayed evolution), and increased fibrosis compared to the EX group, suggesting impaired muscle regeneration and excessive scar-tissue formation. Recently, the positive effects of increased muscle loading through extracorporeal shock waves during SMR—enhancing myogenic-cell number, their proliferation, and differentiation rates—on rat skeletal muscle have been documented [18], and some human studies also showed that increasing muscle tensions through eccentric exercise training has clear effects on the improvement of the skeletal muscle to a favourable and more functional phenotype during SMR [19,20]. Our results suggest that decreasing muscle recruitment during SMR delays and impairs myogenic cells’ ability to cope with injury when compared to the faster and greater myotube formation rate promoted by regular voluntary running. Considering that cluster differentiation (CD) 68 is also expressed by early developing cells, allowing them to attach to selectins during maturation, and in addition, the M1 macrophage identification by CD68, this immunohistochemistry technique also allowed for the categorization of an earlier stage of myotube development. In fact, the increased number of developing myotubes in the SED group suggests a delayed myotube maturation when compared to the EX group, which presented an increased number of myotubes without expressing CD68. Disregarding the lack of studies analysing the effects of increased muscle recruitment on the extracellular matrix dynamics, it is known that both increased voluntary running and exercise influence collagen turnover and matrix metalloproteinase activity [21]. Moreover, others also showed that increased muscle loading successfully decreased fibrosis during SMR [22]. Despite the number of M2 macrophages not differing between groups, the SED group showed increased numbers of both M1 macrophages and neutrophils, i.e., representative inflammatory cells of the Th1 phase. Indeed, over the past few years, the pro-inflammatory environment promoted by sedentary behaviour [23] and the anti-inflammatory effects of regular physical activity on the immune system [24] and in several conditions [25] have been acknowledged, corroborating our results, since sedentary behaviour exacerbated the Th1 phase, increasing the number of pro-inflammatory cells infiltrating the damaged tissue. Additionally, data evincing the effects of pro-inflammatory macrophages in both exacerbating tissue damage and increasing fibrosis have been thoroughly documented [8,9,26]. Thus, is seems appealing to consider that the aggravated Th1 inflammatory response induced by sedentary behaviour significantly impairs both the myotube formation and the fibrotic-tissue accretion. Moreover, considering also the emerging data linking the effects of anti-inflammatory macrophages in both decreasing fibrotic activity and boosting the resolution phase during tissue repair [27], our results suggest that the increased muscle recruitment enhances the Th2 inflammatory response at an early phase of SMR, since the EX group presented an increased number of M2 macrophages on the 1st dpi. In fact, the anti-inflammatory effects of exercise during musculoskeletal disorders have been recently proposed as a mean of therapeutic approach [28]. Furthermore, our results show that this anti-inflammatory effect of muscle recruitment decreases the number of the pro-inflammatory cells needed during SMR, accelerates the myotube maturation, and decreases the fibrotic-tissue accretion.

On the 15th dpi, the SED group presented an increased myotube percentage compared to the EX group—i.e., the SED group showed only 54% of normal undamaged fibres compared to 90% from the EX group. These results strengthen those from the 7th dpi, demonstrating that decreased muscle recruitment effectively delays the myotube formation rate and their maturation process during SMR. Furthermore, the SED group showed, again, increased fibrosis compared to the EX group. These results support those from the 7th dpi, corroborating the detrimental effect inflicted by the reduced muscle recruitment in inducing the fibroblasts to excessively produce fibrotic tissue, culminating in the development of a more unfavourable skeletal-muscle phenotype during SMR. The SED group still showed an increased number of all the inflammatory cells analysed, clearly demonstrating (1) an impaired tissue resolution, i.e., a prolonged accumulation of degenerated and/or necrotic cells and debris with the corresponding increase in the inflammatory infiltrate, and (2) an increased amount of time necessary for proper tissue healing, mainly achieved by scar-tissue formation. Indeed, considering the proposed positive effects of both acute and chronic muscle recruitment on macrophage functions [11,27], our results suggest that sedentary behaviour may also impair this cell function, since the SED group showed an increased number of M1 macrophages on both the 7th and the 15th dpi, demonstrating the need for additional macrophages to accomplish proper debris clearance. Considering the data of the overall fibrosis progression, our results clearly show the regular scar-tissue accretion during SMR. However, in normal physiological circumstances, this increased collagen deposition is usually reversible [29] and an unnecessary or impaired scar-tissue accumulation, like that showed by the SED group, resembles that of pathological conditions [30]. Again, our data evince the negative effect of sedentary behaviour on the overall fibroblast response during SMR.

## 5. Conclusions

The presented data show that sedentary behaviour, through decreased muscle recruitment, has important detrimental effects during SMR, i.e., exacerbating the pro-inflammatory Th1 phase that results in impaired myotube development and fibroblast functionality. Additionally, our results also evince that increased muscle recruitment during SMR has important effects on macrophage response, apparently shortening the inflammatory response and hindering an excessive accumulation of neutrophils. These results support our initial hypothesis that levels of physical activity modulate the tissue inflammatory response and the healing process, with sedentary behaviour negatively affecting skeletal muscles’ ability to repair.

## Figures and Tables

**Table 1:** Histomorphometric data and inflammatory-cell presence in the different groups.

	Histomorphometry						Immunohistochemistry (Cells/mm^2^)					
	Necrotic Fibres (%)		Myotubes (%)		Fibrosis (mm^2^)		Macrophages 1		Macrophages 2		Neutrophils	
Day/Group	SED	EX	SED	EX	SED	EX	SED	EX	SED	EX	SED	EX
1st dpi	86.3	83.7	0.0	0.0	11.7	11.1	0.22	0.32	0.18 *	0.26	0.31 *	0.20
7th dpi	0.0	0.0	58.4 *	69.3	36.1 *	24.9	0.34 *	0.24	0.26	0.25	0.81 *	0.58
15th dpi	0.0	0.0	46.0 *	10.2	17.9 *	8.4	0.17 *	0.00	0.11 *	0.00	0.03 *	0.00

Results of histomorphometry (percentage of necrotic fibres and myotubes, and the area of fibrosis) and immunohistochemistry (number of inflammatory cells per area expressing macrophages 1 and 2 subtypes, and neutrophils) of the muscle-damaged regions from the sedentary (SED) and exercise (EX) groups sacrificed at the 1st, 7th, and 15th day post-injection (dpi). * *p* < 0.01 SED vs. EX groups.

## Data Availability

All datasets used/analysed in the present study are presented in the manuscript.
